# Outcomes of children with hepatoblastoma who underwent liver resection at a tertiary hospital in China: a retrospective analysis

**DOI:** 10.1186/s12887-020-02059-z

**Published:** 2020-05-09

**Authors:** Jiahao Li, Huixian Li, Huiying Wu, Huilin Niu, Haibo Li, Jing Pan, Jiliang Yang, Tianbao Tan, Chao Hu, Tao Xu, Xiaohong Zhang, Manna Zheng, Kuanrong Li, Yan Zou, Tianyou Yang

**Affiliations:** 1 Department of Pediatric Surgery, Guangzhou Women and Children’s Medical Center, Guangzhou Medical University, 9 Jinsui Road, Guangzhou, 510623 Guangdong China; 2 Institute of Pediatrics, Guangzhou Women and Children’s Medical Center, Guangzhou Medical University, Guangzhou, 510623 China; 3 Department of Radiology, Guangzhou Women and Children’s Medical Center, Guangzhou Medical University, Guangzhou, 510623 China; 4 Department of Pathology, Guangzhou Women and Children’s Medical Center, Guangzhou Medical University, Guangzhou, 510623 China; 5 Department of Interventional Radiology, Guangzhou Women and Children’s Medical Center, Guangzhou Medical University, Guangzhou, 510623 China; 6 Department of Hematology/Oncology, Guangzhou Women and Children’s Medical Center, Guangzhou Medical University, Guangzhou, 510623 China

**Keywords:** Hepatoblastoma, Surgery, Children, Liver tumour

## Abstract

**Background:**

To report the outcomes of hepatoblastoma resected in our institution.

**Methods:**

We diagnosed 135 children with hepatoblastoma at our institution between January 2010 and December 2017. Patients who underwent liver resection were included for analysis. However, patients who abandoned treatment after diagnosis were excluded from analysis, but their clinical characteristics were provided in the supplementary material.

**Results:**

Forty-two patients abandoned treatment, whereas 93 patients underwent liver resection and were included for statistical analysis. Thirty-six, 23, 3, and 31 patients had PRETEXT stages II, III, IV, and unspecified tumours, respectively. Seven patients had ruptured tumour; 9 had lung metastasis (one patient had portal vein thrombosis concurrently). Sixteen patients underwent primary liver resection; 22, 25, and 30 patients received cisplatin-based neoadjuvant chemotherapy and delayed surgery, preoperative transarterial chemoembolization (TACE) and delayed surgery, and a combination of cisplatin-based neoadjuvant chemotherapy, TACE, and delayed surgery, respectively. Forty patients had both PRETEXT and POST-TEXT information available for analysis. Twelve patients were down-staged after preoperative treatment, including 2, 8, and 2 patients from stages IV to III, III to II, and II to I, respectively. Ten patients with unspecified PRETEXT stage were confirmed to have POST-TEXT stages II (*n* = 8) and I (*n* = 2) tumours. Seven tumours were associated with positive surgical margins, and 12 patients had microvascular involvement. During a median follow-up period of 30.5 months, 84 patients survived without relapse, 9 experienced tumour recurrence, and 4 died. The 2-year event-free survival (EFS) and overall survival (OS) rates were 89.4 ± 3.4%, and 95.2 ± 2.4%, respectively; they were significantly better among patients without metastasis (no metastasis vs metastasis: EFS, 93.5 ± 3.7% vs 46.7 ± 19.0%, adjusted *p* = 0.002. OS, 97.6 ± 2.4% vs 61.0 ± 18.1%, adjusted *p* = 0.005), and similar among patients treated with different preoperative strategies (chemotherapy only vs TACE only vs Both: EFS, 94.7 ± 5.1% vs 91.7 ± 5.6% vs 85.6 ± 6.7%, *p* = 0.542. OS, 94.1 ± 5.7% vs 95.7 ± 4.3% vs 96.7 ± 3.3%, *p* = 0.845).

**Conclusion:**

The OS for patients with hepatoblastoma who underwent liver resection was satisfactory. Neoadjuvant chemotherapy and TACE seemed to have a similar effect on OS. However, the abandonment of treatment by patients with hepatoblastoma was common, and may have biased our results.

## Background

Hepatoblastoma is tshe most common childhood liver malignancy, and has a prevalence of 1 per 1,000,000 population [1, 2]. The incidence of hepatoblastoma has increased in the past two decades, and this upward trend has been correlated with an increasing survival rate among premature and low-birth-weight infants [3]. Hepatoblastoma usually affects children younger than 3 years, and presents as a large abdominal mass. Some patients may present with sudden abdominal pain and haemorrhagic shock in the scenario of tumour rupture. A combination of elevated α-fetoprotein protein (AFP) level and radiographically identified hepatic mass suffices for the clinical diagnosis of hepatoblastoma in children with ages between 6 months and 3 years. However, biopsy, preferably via ultrasound-guided core needle biopsy is recommended for patients of all age groups [4, 5].

The treatment of hepatoblastoma is multidisciplinary; a combination of platinum-based chemotherapy and complete surgical removal is the mainstay of treatment. Cisplatin-based chemotherapy and surgical resection provide standard-risk patients with a 5-year overall survival (OS) of more than 90% [6, 7]. Primary hepatic resection is recommended for patients with PRETEXT stages I and II tumours with no additional annotative risk factors. Otherwise, patients should undergo neoadjuvant chemotherapy and delayed surgery. Orthotopic liver transplantation is an ideal treatment option for patients with PRETEXT stage IV hepatoblastomas and other forms of unresectable hepatoblastomas, and can provide them with more than 80% 5-year OS in the contemporary era [7–9]. Trans-arterial chemoembolization (TACE) alone, or in combination with high-intensity focused ultrasound, may be considered for those with unresectable tumours that are not responsive to primary systemic chemotherapy and are also not suitable for liver transplantations [10].

Nonetheless, the outcomes of hepatoblastoma in developing countries are still far more inferior to those in developed countries [11]. Treatment abandonment among children with cancer is not an unusual phenomenon in developing countries, particularly among those with advanced stage cancers [12]. Furthermore, patients in developing countries have far more limited access to liver transplantation. In order to improve the management and outcomes of hepatoblastoma in developing countries, such experiences are worth reporting. Herein, we described our experiences in treating hepatoblastoma at a tertiary hospital in South China.

## Methods

The diagnosis of hepatoblastoma was initially made based on an elevated AFP level and radiographic detection of a liver mass, and confirmed via pathological examination of samples obtained via either biopsy or primary liver resection. Only hepatoblastoma patients who underwent liver resection were included for statistical analysis. Patients who abandoned treatment were excluded from further analysis. Patients with hepatocellular carcinoma and other liver malignancies were excluded.

One hundred and thirty-five children were diagnosed with hepatoblastoma at our institution between January 2010 and December 2017. Forty-two cases were excluded from the analysis mainly due to treatment abandonment, including 6 cases who died due to aggressive tumour progression prior to treatment and 36 cases that received no further treatment after diagnosis. The demographic and clinical characteristics of these excluded patients was collected and analysed. Our study analysed 93 cases that were treated according to the institutional protocol and underwent liver resection. Preoperative TACE was optional and available for patients with PRETEXT stage III and IV tumours, after evaluated by the interventional radiologist. The chemotherapy regimens of COG (Children’s Oncology Group), SIOPEL (International Childhood Liver Tumours Strategy Group), and our national regimens were used. All these chemotherapy regiments were cisplatin-based and were reported to have similar effects and achieved similar survival outcomes [13]. Patients were followed up at the clinic and via regular telephone calls. The primary outcome was to evaluate the event-free survival and overall survival of hepatoblastoma resected in our institution. The secondary outcome was to analyse factors that would impact survival in this cohort of patients. The OS duration was defined as the interval between the time of diagnosis and the time of death, and event-free survival (EFS) as the interval between the time of diagnosis and the time of the first occurrence of tumour progression, relapse, or death, whichever occurred first.

We collected information regarding patients’ demographic data, including age and gender; clinical data including AFP level, radiographic findings, pre-treatment extent of tumour (PRETEXT) and post-treatment extent of tumour (POST-TEXT) staging, preoperative management strategy (neoadjuvant chemotherapy and TACE), and liver resection technique; pathological findings including pathological subtype, surgical margin status, microvascular involvement, and lymph node involvement; and clinical outcomes including disease relapse and death.

A standard data extraction form with a logical organisation similar in flow to the format of the original medical charts, was used to collect data. Two trained data abstractors, who were blinded to the study hypothesis, independently reviewed the original medical charts and collected data. Explicit criteria for extracting data regarding variables were applied. Any discrepancies between the abstractors were reviewed jointly and discussed to clarify any issues [14].

A senior radiologist, who was blinded to the study objective, retrospectively reviewed patients’ computed tomography (CT) and magnetic resonance imaging (MRI) data. The radiologist defined the PRETEXT/POST-TEXT system and annotation factors according to the PRETEXT staging system [15]. Not all patients had CT/MRI images stored in the electronic database; only patients who underwent CT/MRI scans at our institution had their radiographic images stored.

The study protocol was approved by the institutional review board of Guangzhou Women and Children’s Medical Centre. The need for informed consent was waived on account of the retrospective nature of the demographic, clinical, and outcome data. All patients’ data were de-identified prior to the analysis.

### Statistical analysis

Categorical variables are presented as numbers and percentages. Continuous variables are presented as medians and ranges. The PRETEXT and POST-TEXT stages were compared using the McNemar chi-square test. The comparison of different management strategies was analysed using the Wilcoxon signed-rank test. The probabilities of OS and EFS were computed using the Kaplan-Meier method and compared using the log-rank test. Statistical significance was set at *p* < 0.05 and *p*-values of the paired tests in the log-rank test were adjusted using the Bonferroni method. All statistical analyses were performed using SAS 9.4 for Windows (SAS Institute Inc., Cary, NC, USA).

## Results

### Patients’ demographic and clinical characteristics

Of the 93 patients who underwent liver resection, 66 (60.2%) were male and 37 (39.8%) were female (Table 1). The median age at diagnosis was 11 (range, 1.7–87) months. The median AFP level was 76,131 (range, 10–1,881,360) ng/ml and the median tumour diameter was 10.6 (range, 5.1–15.8) cm. Fifty-seven (61.3%) patients had unifocal tumours, 7 (7.5%) had multifocal tumours, and 29 (31.2%) had tumours with unspecified focality.

**Table 1:** Demographic, clinical, radiological, and pathological characteristics of the study cohort

Characteristics	Number or as shown	Proportion (%)
All	93	100
Gender		
Male	56	60.2
Female	37	39.8
Age [median (range)], months	11 (1.7–87)	–
AFP level [median (range)], ng/ml	76,131 (10–1,881,360)	–
Maximum tumour diameter [median (range)], cm	10.6 (5.1–15.8)	–
Focality		
Unifocal	57	61.3
Multifocal	7	7.5
Unknown	29	31.2
PRETEXT stage		
I	0	0.0
II	36	38.7
III	23	24.7
IV	3	3.2
Unknown	31	33.3
Rupture		
Yes	7	7.5
No	56	60.2
Unknown	30	32.3
Metastasis		
Yes	9	9.7
No	55	59.1
Unknown	29	31.2
Portal vein thrombosis		
Yes	1	1.1
No	63	67.7
Unknown	29	31.2
Hepatic vein thrombosis		
Yes	0	0.0
No	64	68.8
Unknown	29	31.2
Primary resection		
Yes	16	17.2
No	77	82.8
Neoadjuvant chemotherapy		
Yes [n, median (range)]	52, 2.5 (1–8)	55.9
No	41	44.1
Preoperative TACE, cycles		
Yes [n, median (range)]	55, 2 (1–7)	59.1
No	38	40.9
POSTTEXT^a^ stage (*n* = 77)		
I	4	5.2
II	36	46.8
III	9	11.7
IV	1	1.3
Unknown	27	35.1

^a^Sixteen children underwent primary tumour resection (with no neoadjuvant chemotherapy and no preoperative TACE), and did not need to undergo POST-TEXT stage evaluation. Abbreviations: *AFP* alpha-fetoprotein, *PRETEXT* pre-treatment extent of disease system, *TACE* transarterial chemoembolisation, *POST-TEXT* post-treatment extent of disease system

Thirty-six (38.7%) patients had PRETEXT stage II tumours, 23 (24.7%) had stage III tumours, 3 (3.2%) had stage IV tumours, and 31 (33.3%) had tumours with unspecified PRETEXT stages. Seven (7.5%) patients had ruptured tumours. Nine patients (9.7%) had lung metastasis, three of them had single lung metastasis and 6 had multiple lung metastasis [1 (1.1%) had portal vein thrombosis concurrently]. Sixteen (17.2%) patients underwent primary liver resection. Twenty-two patients (23.7%) received cisplatin-based neoadjuvant chemotherapy and delayed surgery, 25 (26.9%) received preoperative TACE and delayed surgery, and 30 (32.3%) received a combination of cisplatin-based neoadjuvant chemotherapy, TACE, and delayed surgery. PRETEXT stage distribution of each treatment group was provided in supplementary Table 1. The median number of treatment cycles was 2.5 (range, 1–8) for neoadjuvant chemotherapy and 2 (range, 1–7) for preoperative TACE. Forty patients had information regarding both PRETEXT and POST-TEXT stages available for analysis. Using the McNemar test, significant downstage was noted for the 12 cases with both PRETEXT and POST-TEXT stage information (*p* < 0.001). Specifically, 2 cases from stage IV to III, 8 from stage III to II, and 2 from stage II to I. Furthermore, 10 patients with unspecified PRETEXT stage were confirmed to have POST-TEXT stages II (*n* = 8) and I (*n* = 2) tumours.

The detailed demographic and clinical characteristics of the excluded 42 patients were listed in supplementary Table 2. The excluded patients were significantly higher in age, AFP value, and PRETEXT stage than the included 93 patients. Additionally, more patients of the excluded group had lung metastases and portal vein thrombosis. The overall outcomes of these patients were largely unknown, and these patients were excluded from further analysis.

### Surgery and outcomes

Thirty-seven (39.8%) patients underwent hemihepatectomy, 17 (18.3%) underwent wedge resection, 13 (14.0%) underwent trisectionectomy, 9 (9.7%) underwent bisegmentectomy (left lateral sectionectomy), and 2 (2.2%) underwent central hepatectomy (Table 2). Fifteen patients underwent liver resection at other institutions, but detailed surgical information was not available. Seventy-eight patients were operated in our institution, and surgical information was collected and analysed. The operative time, estimated volume of blood lost, and volume of red blood cells transfused were 290 (range, 100–510) minutes, 8.9 (range, 1.7–111.1) ml/kg, and 26.7 (range, 0–111.1) ml/kg, respectively. There were 24 (25.8%) cases of epithelial variant hepatoblastoma, 11 (11.8%) cases of mixed epithelial hepatoblastoma, and 41 (44.1%) cases of mixed epithelial and mesenchymal hepatoblastoma; 17 cases were not sub-classified. Seven (7.5%) cases had positive surgical margins, 69 (74.2%) had negative surgical margins, and 17 (18.3%) had unspecified surgical margin status. Twelve (12.9%) patients had microvascular involvement, 43 (46.2%) had no microvascular involvement, and 38 (40.9%) cases had unspecified microvascular status. Thirty-one patients underwent lymph node dissection, none of whom had positive lymph node involvement. Among the 9 patients with lung metastasis, one underwent metastasectomy.

**Table 2:** Surgical and pathological outcomes of patients managed for hepatoblastoma

Characteristics	Number or as shown	Proportion (%)
Liver resection		
Hemihepatectomy (left hepatectomy + right hepatectomy)	37	39.8
Wedge resection	17	18.3
Trisectionectomy (left trisectionectomy + right trisectionectomy)	13	14.0
Bisegmentectomy (left lateral sectionectomy)	9	9.7
Central hepatectomy	2	2.2
Others	15	16.1
Operative time [median (range)], minutes	290 (100–510)	–
Estimated blood loss [median (range)], ml/kg	8.9 (1.7–111.1)	–
Volume of red blood cells transfused [median (range)], ml/kg	26.7 (0–111.1)	–
Pathologic subtype	93	
Epithelial variants	24	25.8
Pure foetal variant with low mitotic activity	3	–
Foetal variant, mitotically active	10	–
Unspecified	11	–
Epithelial mixed	11	11.8
Mixed epithelial and mesenchymal	41	44.1
Without teratoid features	2	–
With teratoid features	15	–
Unspecified	24	–
Unknown	17	18.3
Surgical margin		
Positive	7	7.5
Negative	69	74.2
Unknown	17	18.3
Microvascular involvement		
Yes	12	12.9
No	43	46.2
Unknown	38	40.9
Lymph node status (*n* = 31)		
Positive	0	0.0
Negative	31	100.0
Postoperative chemotherapy		
Yes [n, median (range)]	63, 6 (1–12)	67.7
No	27	29.0
Unknown	3	3.2
Outcomes		
Survived without relapse	84	90.3
Survived with relapse	5	5.4
Died from relapse	4	4.3
Median follow-up duration [median (range)], months	30.5 (0.7–105.1)	–

The operative time, estimated volume of blood lost, and volume of red blood cells transfused were calculated based on 78 patients operated in our institution

Sixty-three (67.7%) patients received cisplatin-based postoperative chemotherapy, with a median of 6 (range, 1–12) cycles. Twenty-seven (29.0%) patients received no postoperative chemotherapy. During a median follow-up duration of 30.5 (range, 0.7–105.1) months, 84 (90.3%) cases survived without relapse, 9 (9.7%) experienced disease recurrence, and 4 (5.4%) died. For the 9 patients with lung metastasis, 5 of them survived with metastasis cleared, 1 died, and 3 were lost to follow-up.

### Subgroup analysis of managements

In this study, the differences in management between patients without metastasis and patients with metastasis (1 of them had portal vein thrombosis at the same time) [cycle of neoadjuvant chemotherapy: 1(0–6) vs 2(0–8), *p* = 0.060; cycle of preoperative TACE: 0(0–5) vs 1(0–7), *p* = 0.589; cycle of postoperative chemotherapy: 6(0–12) vs 6(2–10), *p* = 0.817], and patients with negative surgical margin and positive surgical margins [cycle of neoadjuvant chemotherapy: 1(0–8) vs 1(0–3), *p* = 0.482; cycle of preoperative TACE: 1(0–5) vs 2(0–7), *p* = 0.081; cycle of postoperative chemotherapy: 6(0–12) vs 7(2–12), *p* = 0.946] were not statistically significant.

### Failure among patients with tumour recurrence

Among the 9 patients with tumour recurrence, the median time from diagnosis to recurrence was 8.5 (range, 0.7–22.4) months, and the median time from surgery to recurrence was 3.6 (range, 0.5–22.0) months. Among the 4 patients who died as a result of tumour recurrence, the median time from diagnosis to death was 11.3 (range, 3.6–21.4) months. Their treatment and outcome information are summarised in Table 3. Five patients underwent wedge resection, and 1 underwent left hepatectomy associated with a positive surgical margin.

**Table 3:** Detailed information of patients who experienced tumour relapse or death

Characteristics	Patients^a^								
	P1	P2	P3	P4	P5	P6	P7	P8	P9
Age, months	7	24	9	5	19	41	46	6	87
AFP at diagnosis	50,000	10.6	24,200	80,000	252.5	80,000	1,000,000	82,480	5000.08
PRETEXT stage	II	III	II	II	IV	II	Null	Null	II
Multifocal tumour	No	No	No	No	Yes	No	Null	Null	No
Metastasis	Yes	Yes	No	Yes	Yes	No	Null	Null	No
Neoadjuvant chemotherapy, cycles	0	2	0	8	4	4	2	0	0
Preoperative TACE	0	2	3	0	7	1	4	5	0
POSTTEXT stage	–	III	II	II	III	II	Null	Null	–
Surgical margin status^b^	N^−^	P^+^	N^−^	N^−^	P^+^	N^−^	Null	Null	N^−^
Postoperative pathologic subtype^c^	Foetal	With TF	EV	MEM	EV	EM	Null	Null	EM
Postoperative chemotherapy, cycles	3	0	6	4	2	Null	Null	4	4
Relapse site	lung	lung	lung	liver, lung	liver, lung	lung	liver	liver	liver
Death	Yes	Yes	Yes	Yes	No	No	No	No	No
Time from diagnosis to death, months	7.7	3.6	21.4	14.8	–	–	–	–	–

^a^null, unknown; −, no need to fill in; ^b^*N*^*−*^ negative, *P*^*+*^ positive, ^c^*EV* epithelial variant, *With TF* with teratoid features, *MEM* mixed epithelial and mesenchymal, *EM* epithelial mixed

### Survival

**Fig. 1:**
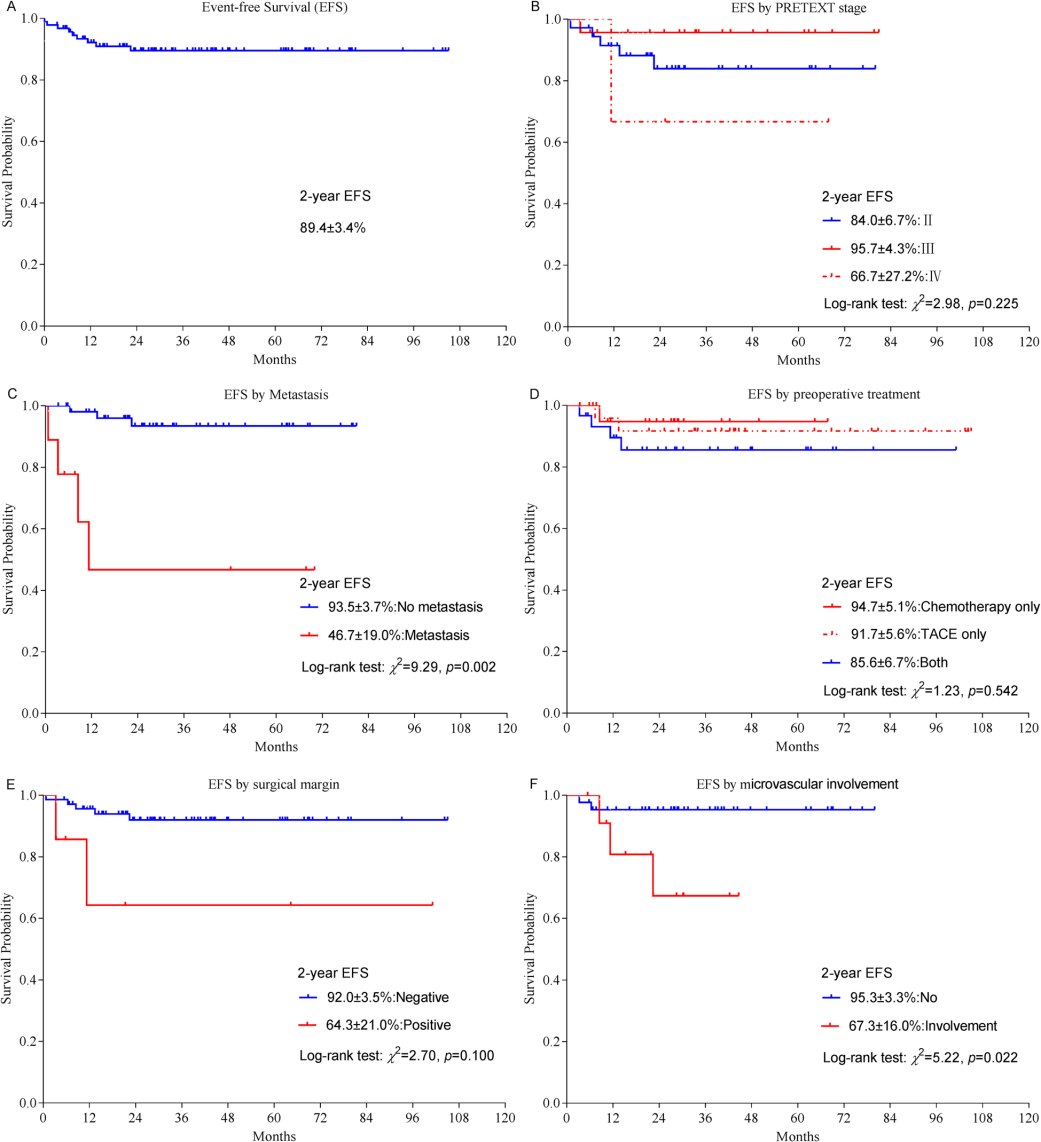
Kaplan-Meier estimates of event-free survival probabilities

**Fig. 2:**
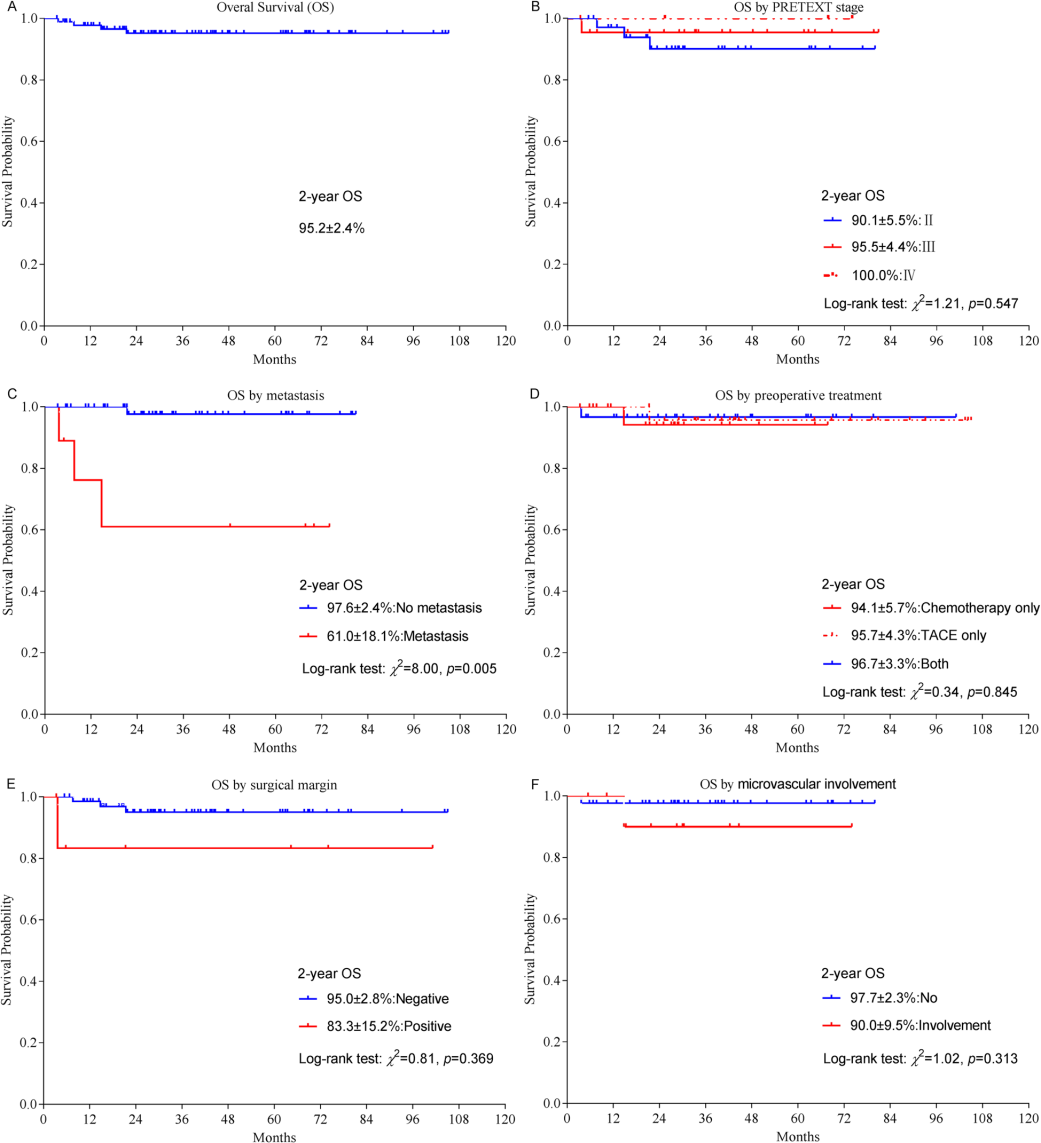
Kaplan-Meier estimates of overall survival probabilities

The 2-year event-free survival (EFS) and overall survival (OS) rates were 89.4 ± 3.4%, and 95.2 ± 2.4% (Figs. 1a and 2a), respectively. The 2-year EFS and OS rates were significantly better among patients without metastasis (no metastasis vs metastasis: EFS, 93.5 ± 3.7% vs 46.7 ± 19.0%, *p* = 0.002, OS, 97.6 ± 2.4% vs 61.0 ± 18.1%, *p* = 0.005) (Figs. 1c and 2c). The 2-year EFS rates were significantly better among patients without microvascular involvement (No vs Involvement: EFS, 95.3 ± 3.3% vs 67.3 ± 16.0%, *p* = 0.022), while the 2-year OS rates were similar (OS, 97.7 ± 2.3% vs 90.0 ± 9.5%, *p* = 0.313). The differences of the 2-year EFS and OS rates of patients with PRETEXT stage IV hepatoblastoma (II vs III vs IV: EFS, 84.0 ± 6.7% vs 95.7 ± 4.3% vs 66.7 ± 27.2%, *p* = 0.225. OS, 90.1 ± 5.5% vs 95.5 ± 4.4% vs 100.0%, *p* = 0.547), positive surgical margins (negative vs positive: EFS, 92.0 ± 3.5% vs 64.3 ± 21.0%, *p* = 0.100. OS, 95.0 ± 2.8% vs 83.3 ± 15.2%, *p* = 0.369) were not statistically significant. The 2-year EFS and OS rates were also similar among patients treated with different preoperative strategies (Chemotherapy only vs TACE only vs Both: EFS, 94.7 ± 5.1% vs 91.7 ± 5.6% vs 85.6 ± 6.7%, *p* = 0.542. OS, 94.1 ± 5.7% vs 95.7 ± 4.3% vs 96.7 ± 3.3% *p* = 0.845) (Figs. 1d and 2d).

## Discussion

Here, we reported the outcomes of resected hepatoblastoma at a tertiary children’s institution in a developing country. The 2-year EFS and OS rates among patients who underwent hepatic resection were satisfactory. Patients associated with distant metastasis had a worse prognosis, with 2-year EFS and OS rates of about 46.7 ± 19.0% and 61.0 ± − 18.1%, respectively. Neoadjuvant chemotherapy and TACE seem to have similar effects on the 2-year EFS and OS.

Both cisplatin-based neoadjuvant chemotherapy and preoperative TACE were used at our institution as preoperative strategies to shrink the tumour and downstage the tumour [16]. However, our results showed no significant differences regarding the effect of neoadjuvant chemotherapy and TACE on 2-year EFS and OS. Similarly, evidence from the Japanese Study Group for Paediatric Liver Tumour (JPLT) and our institution showed that TACE was as effective as neoadjuvant chemotherapy in shrinking and down-staging tumours [16, 17]. However, the JPLT study showed that the OS was inferior to that of those who underwent neoadjuvant chemotherapy [17]. TACE could be an option for patients who fail to respond to neoadjuvant chemotherapy. Furthermore, TACE is particularly useful for patients who experience tumour rupture [18]. Currently, neoadjuvant chemotherapy is considered the first choice for the preoperative management of hepatoblastoma. However, no prospective study has compared the effect of neoadjuvant chemotherapy and TACE on hepatoblastoma. It would be valuable to compare these two strategies in a prospective or randomized trial.

Patients with tumour metastasis had significantly lower 2-year EFS and OS. The 2-year EFS and OS for patients with metastatic disease were only about 46.7 ± 19.0% and 61.0 ± 18.1%, respectively. Our result was consistent with the SIOPEL experiences, which showed that hepatoblastoma with metastasis has a 3-year EFS of 49% [19]. However, we failed to demonstrate that patients with PRETEXT stage IV tumours had significantly worse EFS and OS probabilities than those with tumours of other stages. However, our cohort only had 3 cases with PRETEXT stage IV tumours. Two cases were down-staged to POST-TEXT stage III, and the other died.

The 2-year EFS and OS for patients with positive surgical margins were lower than those of their counterparts, but the differences were not statistically significant. The evidence suggested that positive surgical margin might not affect the EFS and OS in the setting of neoadjuvant chemotherapy [20]. However, this might not be true in the setting of primary resection. Complete resection with a negative resection margin should always be pursued. Microvascular involvement was suggested to be a poor prognostic factor in a retrospective study [21]. In our cohort, 12 (12.9%) patients had microvascular involvement, 43 patients had no microvascular involvement, and 38 patients had tumours with unspecified microvascular status. Our data suggested that patients with microvascular involvement had significant lower 2-year EFS than those without microvascular involvement, but the OS were similar between the two groups. Again, in the current Children’s Hepatic tumours International Collaboration classification system, microvascular involvement is not considered as a risk factor [6, 22].

Hepatoblastoma seemed not to spread through the lymph nodes. None of the 31 patients who underwent lymph node biopsy had positive lymph node involvement.

Five out of 9 patients who experienced relapse or died underwent wedge resection. This suggests that wedge resection might be associated with worse outcomes. Standard hepatic resection should always be pursued in any possible scenario.

Due to the retrospective nature of this study, we were unable to retrieve some of the important information. For example, some of the patients did not undergo preoperative CT or MRI scans for PRETEXT staging. Furthermore, a large proportion of the patients abandoned or discontinued treatment after the establishment of the diagnosis. These patients will most likely fall into the high-risk group (Supplemental Table 2). In fact, the excluded patients were significantly higher in age and PRETEXT stage than included patients. Among the excluded patients, more patients had metastasis and portal vein thrombosis. Overall, the excluded patients mostly had advanced stage hepatoblastoma, and would have much worse survival. Unfortunately, we were not able to follow these excluded patients. The exclusion of these patients will incur selection bias. Treatment abandonment is not an unusual phenomenon in developing countries, which underscores the need for more attention and funding for this vulnerable population [23, 24]. Furthermore, the follow-up duration was not long enough, and the EFS and OS might either be overestimated if patients abandoned treatment due to poor results, or underestimated if patients abandoned treatment because their parents prematurely assumed they were cured. An assessment of the interactions between different characteristics requires more stable follow-up with larger samples.

## Conclusions

The overall outcomes for those who underwent liver resection was satisfactory. However, the abandonment of treatment by patients with hepatoblastoma was common. A large proportion of patients discontinued treatment after the diagnosis.

## Supplementary information

**Additional file 1: Table S1.** Pretext stage distribution of different treatment strategies.

**Additional file 2: Table S2.** Comparison of demographic, clinical, radiological, and pathological characteristics between included and excluded patients.

## Data Availability

The datasets generated and/or analysed during the current study are not publicly available due to patient privacy but are available from the corresponding author on reasonable request.

## Abbreviations

AFP: Alpha-fetoprotein
CT: Computed tomography
CHIC: Children’s Hepatic tumours International Collaboration
COG: Children’s Oncology Group
EFS: Event-free survival
JPLT: Japanese Study Group for Pediatric Liver Tumour
MRI: Magnetic resonance imaging
OS: Overall survival
PRETEXT: Pre-treatment extent of tumour
POST-TEXT: Post-treatment extent of tumour
SIOPEL: International Childhood Liver Tumours Strategy Group
TACE: Transarterial chemoembolisation
