# Supplementary material for: Outcomes of children with hepatoblastoma who underwent liver resection at a tertiary hospital in China: a retrospective analysis
Source: BMC Pediatr. 2020 May 9;20:200. doi: 10.1186/s12887-020-02059-z (PMC7210686; doi:10.1186/s12887-020-02059-z)
Supplement: Supplementary file 1 — Additional file 1: Table S1. Pretext stage distribution of different treatment strategies. [file 12887_2020_2059_MOESM1_ESM.docx]

Supplementary Table 1. Pretext stage distribution of different treatment strategies.

|  | N (%) |
| --- | --- |
| Primary surgery, n=16 |  |
| Pretext I | 0(0) |
| Pretext II | 10(62.5) |
| Pretext III | 2(12.5) |
| Pretext IV | 0(0) |
| Unknown | 4(25.0) |
| Neoadjuvant chemotherapy + surgery, n=22 |  |
| Pretext I | 0(0) |
| Pretext II | 14(63.6) |
| Pretext III | 5(22.7) |
| Pretext IV | 1(4.5) |
| Unknown | 2(9.1) |
| TACE + surgery, n=25 |  |
| Pretext I | 0(0) |
| Pretext II | 2(8.0) |
| Pretext III | 11(44.0) |
| Pretext IV | 1(4.0) |
| Unknown | 11(44.0) |
| Neoadjuvant chemotherapy + TACE + surgery, n=30 |  |
| Pretext I | 0(0) |
| Pretext II | 10(33.3) |
| Pretext III | 5(16.7) |
| Pretext IV | 1(3.3) |
| Unknown | 14(46.7) |
