# Supplementary material for: Outcomes of children with hepatoblastoma who underwent liver resection at a tertiary hospital in China: a retrospective analysis
Source: BMC Pediatr. 2020 May 9;20:200. doi: 10.1186/s12887-020-02059-z (PMC7210686; doi:10.1186/s12887-020-02059-z)
Supplement: Supplementary file 2 — Additional file 2: Table S2. Comparison of demographic, clinical, radiological, and pathological characteristics between included and excluded patients. [file 12887_2020_2059_MOESM2_ESM.doc]

Supplemental Table 2. Comparison of demographic, clinical, radiological, and pathological characteristics between included and excluded patients.

| Characteristics | Included | |  | Excluded | | *P* value |
| --- | --- | --- | --- | --- | --- | --- |
|  | Number or as shown | Proportion (%) |  | Number or as shown | Proportion (%) |  |
| All | 93 | 100 |  | 43 | 100 | - |
| Gender |  |  |  |  |  | 0.552 |
| Male | 56 | 60.2 |  | 23 | 54.8 |  |
| Female | 37 | 39.8 |  | 19 | 45.2 |  |
| Age [median (range)], months | 11 (1.7–87) | - |  | 17(0.6-140.9) | - | 0.010^*^ |
| AFP level [median (range)], ng/ml | 76,131 (10–1,881,360) | - |  | 158577(133.1-880800) | - | 0.002^*^ |
| Maximum tumour diameter [median (range)], cm | 10.6 (5.1–15.8) | - |  | 11.7(5.9-17.3) | - | 0.230^*^ |
| Focality |  |  |  |  |  | 0.276 |
| Unifocal | 57 | 61.3 |  | 21 | 50.0 |  |
| Multifocal | 7 | 7.5 |  | 2 | 4.8 |  |
| Unknown | 29 | 31.2 |  | 19 | 45.2 |  |
| PRETEXT stage |  |  |  |  |  | 0.043 |
| I | 0 | 0.0 |  | 1 | 2.4 |  |
| II | 36 | 38.7 |  | 6 | 14.3 |  |
| III | 23 | 24.7 |  | 14 | 33.3 |  |
| IV | 3 | 3.2 |  | 2 | 4.8 |  |
| Unknown | 31 | 33.3 |  | 19 | 45.2 |  |
| Rupture |  |  |  |  |  | 0.332 |
| Yes | 7 | 7.5 |  | 2 | 4.8 |  |
| No | 56 | 60.2 |  | 21 | 50.0 |  |
| Unknown | 30 | 32.3 |  | 19 | 45.2 |  |
| Metastasis |  |  |  |  |  | 0.017 |
| Yes | 9 | 9.7 |  | 12 | 28.6 |  |
| No | 55 | 59.1 |  | 18 | 42.9 |  |
| Unknown | 29 | 31.2 |  | 12 | 28.5 |  |
| Portal vein thrombosis |  |  |  |  |  | 0.008 |
| Yes | 1 | 1.1 |  | 4 | 9.5 |  |
| No | 63 | 67.7 |  | 19 | 45.2 |  |
| Unknown | 29 | 31.2 |  | 19 | 45.2 |  |
| Hepatic vein thrombosis |  |  |  |  |  | 0.080 |
| Yes | 0 | 0.0 |  | 1 | 2.4 |  |
| No | 64 | 68.8 |  | 22 | 52.4 |  |
| Unknown | 29 | 31.2 |  | 19 | 45.2 |  |

PRETEXT, pre-treatment extent of disease system; TACE, transarterial chemoembolization; ^*^The differences were compared by Wilcoxon signed-rank tests, and the others were compared by Chi-squared test.
